
JOURNAL INFORMATION
==============================
NLM Title Abbreviation: Medizinrecht
Journal ID: Medizinrecht
NLM Journal ID: 8309354

Medizinrecht

ISSN: 0723-8886
EISSN: 1433-8629

ARTICLE INFORMATION
==============================
PMCID: PMC9709745
PMID: 36467114
DOI: 10.1007/s00350-022-6331-6
Article ID: 6331
Article version: 1
Subjects: Aufsätze

Pandemic Preparedness bei der Obduktion? Potentiale und Grenzen des IfSG

Gaede Karsten 1
Heidemann John LL.M. oec 1
Goebels Hanna-Lisa 2
Heinemann Axel 2
Püschel Klaus 2
Ondruschka Benjamin 2
1 grid.461688.5 0000 0000 9215 6192 Institut für Medizinrecht, Bucerius Law School, Hamburg, Deutschland
2 grid.13648.38 0000 0001 2180 3484 Institut für Rechtsmedizin, UKE Hamburg, Hamburg, Deutschland
Electronic publication date: 2022 Nov 30
Print publication date: 2022
Volume: 40
Issue: 11
First page: 892
Last page: 901
Copyright: © Der/die Autor(en) 2022
License: Open Access Dieser Artikel wird unter der Creative Commons Namensnennung 4.0 International Lizenz veröffentlicht, welche die Nutzung, Vervielfältigung, Bearbeitung, Verbreitung und Wiedergabe in jeglichem Medium und Format erlaubt, sofern Sie den/die ursprünglichen Autor(en) und die Quelle ordnungsgemäß nennen, einen Link zur Creative Commons Lizenz beifügen und angeben, ob Änderungen vorgenommen wurden.
Die in diesem Artikel enthaltenen Bilder und sonstiges Drittmaterial unterliegen ebenfalls der genannten Creative Commons Lizenz, sofern sich aus der Abbildungslegende nichts anderes ergibt. Sofern das betreffende Material nicht unter der genannten Creative Commons Lizenz steht und die betreffende Handlung nicht nach gesetzlichen Vorschriften erlaubt ist, ist für die oben aufgeführten Weiterverwendungen des Materials die Einwilligung des jeweiligen Rechteinhabers einzuholen.
Weitere Details zur Lizenz entnehmen Sie bitte der Lizenzinformation auf http://creativecommons.org/licenses/by/4.0/deed.de.
License URL: https://creativecommons.org/licenses/by/4.0/

issue-copyright-statement: © Springer-Verlag GmbH Deutschland, ein Teil von Springer Nature 2022

==============================

**Karsten Gaede, John Heidemann, Hanna-Lisa Goebels, Axel Heinemann, Klaus Püschel und Benjamin Ondruschka**

Als der COVID-19-Ausbruch sich zu einer offiziellen Pandemie ausweitete, stand unsere Rechtsordnung vor der Grundfrage, wie der Schutz vor einer auch lebensgefährlichen Infektion unter einer weithin fehlenden Faktenkenntnis mit den Grundrechten ausgeglichen werden sollte. Bekanntlich war insbesondere ein so nie dagewesener Lockdown die möglicherweise wiederkehrende Antwort.

Vor allem wissenschaftlichen Erkenntnissen und Innovationen ist es zu verdanken, dass wir unsere Freiheit heute mit deutlich abgeschwächten Schutzmaßnahmen wieder ausleben können. Schon in der dunklen Anfangszeit der Pandemie waren Hoffnungsschimmer in Deutschland insbesondere einer international viel beachteten Obduktionsserie zu entnehmen, die unter der Federführung von Prof. Dr. med. *Klaus Püschel* am Institut für Rechtsmedizin des Universitätsklinikums Hamburg-Eppendorf (UKE) entstanden war[1]. Durch die Anordnung systematischer Obduktionen[2] corona-assoziierter Todesfälle nach §25 Abs. 1, 4 S. 2 IfSG konnten Erkenntnisse hinsichtlich der Pathologie und dem spezifischen Gewebetropismus des SARS-CoV-2-Virus gewonnen werden[3]. Sie führten u.a. zu einer intensivierten Thromboembolieprophylaxe bei der Behandlung von COVID-19-Erkrankten. Risikofaktoren für einen schwerwiegenden Krankheitsverlauf wurden erkannt[4], was spezifischere Schutzmaßnahmen für vulnerable Personengruppen denkbar werden ließ. Dieser sog. Hamburger Weg wurde im NUM, dem später vom Bundesministerium für Bildung und Forschung (BMBF) zur akademischen Pandemieabwehr gegründeten deutschen Netzwerk Universitätsmedizin als vorbildhaft empfunden; seine deutschlandweite Etablierung als Mittel der seit 2020 angestrebten *pandemic preparedness* wurde untersucht und zum Ziel erklärt[5].

Sind wir dementsprechend gut für die uns nun nicht mehr nur theoretisch erscheinende nächste Pandemie aufgestellt? Werden wissenschaftliche Höchstleistungen erneut zügig sowohl Menschenleben als auch Freiheitsrechte schützen? Leider ist hier zu zeigen, dass die *pandemic preparedness* im Kontext der Obduktion auch aus Rechtsgründen bisher fehlt. Zunächst wird die Rechtsgrundlage der Amtsobduktion und ihre Evolution im Pandemieverlauf vorgestellt (I.). Es wird verdeutlicht, welche Hürden einer bundesweit überzeugenden Obduktionspraxis im Pandemiekontext entgegenstehen (II.). Dies führt dazu, die Reichweite der zulässigen Amtsobduktion zu analysieren und zu bewerten (III.). Daraus sind Schlussfolgerungen zu ziehen, die den tatsächlichen, aber auch rechtlichen Handlungsbedarf umreißen (IV.).

Prof. Dr. iur. Gaede, geschäftsführender Direktor des Instituts für Medizinrechts der Bucerius Law School, Hamburg, Deutschland

John Heidemann LL.M. oec, wissenschaftlicher Mitarbeiter am Institut für Medizinrecht der Bucerius Law School, Hamburg, Deutschland

PD Dr. med. Axel Heinemann, Institut für Rechtsmedizin des UKE Hamburg, Deutschland

Prof. Dr. med. Klaus Püschel, ehemaliger Direktor des Instituts für Rechtsmedizin des UKE Hamburg, Deutschland

Prof. Dr. med. Benjamin Ondruschka, Direktor des Instituts für Rechtsmedizin des UKE Hamburg, Deutschland

## I. Grundlagen des geltenden Rechts (§25 IfSG)

Die Gesetzgebungskompetenz für das Sektionswesen liegt grundsätzlich bei den Ländern[6]. Die Zweckbestimmungen einzelner Sektionsarten berühren jedoch Kompetenzen des Bundes. Von dieser Kompetenz hat der Bund für den Infektionsschutz gemäß Art. 74 Abs. 1 Nr. 19 GG mit §25 Abs. 4 S. 2 im vierten Abschnitt des IfSG Gebrauch gemacht, der sich der „Bekämpfung übertragbarer Krankheiten“ widmet.

Der tradierten und im Wortlaut auf die früheren §§31 und 32 BSeuchG zurückgehenden Vorschrift[7], kam in der Praxis bisher kaum reale Bedeutung zu. Gemäß §25 Abs. 4 S. 2 IfSG soll die zuständige Behörde gegenüber dem Gewahrsamsinhaber eines Verstorbenen die innere Leichenschau anordnen, wenn dies vom Gesundheitsamt für erforderlich gehalten wird. Die innere Leichenschau umfasst die Leichenöffnung zur Feststellung der Todesursache und/oder zur Rekonstruktion des Sterbevorgangs[8].

### *1. Maßnahmenadressat und Grundrechtssensibilität*

Als Maßnahmenadressat ist wortlautgetreu der jeweilige Gewahrsamsinhaber über den Leichnam anzusehen und damit derjenige, der die unmittelbare tatsächliche Sachherrschaft über die Leiche ausübt[9]. Er allein ist zur Duldung der Ermittlungsmaßnahme fähig.

Zugleich darf nicht übersehen werden, dass die Anordnung einer Obduktion nach §25 Abs. 4 S. 2 IfSG auch im

1) Als Beispiele für zahlreiche international beachtete Publikationen s. *Edler/Schröder et al.*, International Journal of Legal Medicine, 2020, 134, 1275ff. sowie *Heinrich/Sperhake/Heinemann/Mushumba/Püschel et al.*, Virchows Archiv, 2020, 477, 335ff.
2) Im Rahmen dieses Beitrags werden die Bezeichnungen „innere Leichenschau“, „Autopsie“, „Obduktion“ und „Sektion“ synonym verwendet, s. *Pschyrembel* (https://www.pschyrembel.de/Sektion/K0KPW/doc/); zur synonymen Verwendung durch die Rechtswissenschaft *Pollähne*, in: *Wienke/Rothschild/Janke*, Rechtsfragen der Obduktion und postmortalen Gewebespende, 2012, S. 33, 34, Fn. 4 m.w.N. Internetquellen wurden zum 17.10.2022 zuletzt aufgerufen.
3) *Wichmann/Sperhake et al.*, Annals of Internal Medicine, 2020, 173, 268ff. sowie *Goebels et al.*, Obduktionen bei COVID-19, klinikarzt, 2020, 49, 404ff.
4) Vgl. *Püschel/Aepfelbacher*, DÄBl., 2020, 117, A 1058ff.
5) Zur Förderung des Hamburger Weges innerhalb von NUM s. die PM des UKE v. 1.10.2020, https://www.uke.de/allgemein/presse/pressemitteilungen/detailseite_99588.html.
6) Vgl. BT-Dr. 13/4355, S. 16; *Lipp*, in: *Laufs/Katzenmeier/Lipp*, Arztrecht, 8. Aufl. 2021, VI, Rdnr. 82.
7) Zur sog. Seuchensektionen bereits §31 Abs. 1 i.V. mit §32 Abs. 3 S. 2 BSeuchG i.d.F. v. 18.12.1979. Zu späteren Umgestaltungen BT-Dr. 14/2530, S. 74 und BT-Dr. 17/7576, S. 33.
8) *Schliephorst*, in: *Wienke/Rothschild/Janke*, Rechtsfragen der Obduktion und postmortalen Gewebespende, 2012, S. 23; *Pschyrembel*, Eintrag „Sektion“, https://www.pschyrembel.de/Sektion/K0KPW/doc/.
9) *Kießling*, in: *Kießling*, IfSG, 3. Aufl. 2022, §25, Rdnr. 25; *Gerhardt*, IfSG, 6. Aufl. 2022, §25, Rdnrn. 15, 55.

Übrigen eine sog. hoheitliche Sektion betrifft[10]. Sie beruht nicht auf einer konsensualen Legitimation, sondern auf einer ggf. gegen den Willen etwaiger Betroffener durchzusetzenden Anordnung von Amts wegen[11]. Bleibt eine Zustimmung aus, stellt die Zwangssektion in jedem Falle einen Grundrechtseingriff dar[12], der je nach Interpretation der Verfassung das postmortale Persönlichkeitsrecht[13] und ggf. die Glaubens- und Bekenntnisfreiheit des Verstorbenen (Art. 4 Abs. 1 GG)[14] als Drittbetroffenem beeinträchtigen kann. Ferner kann die Sektion das Totenfürsorgerecht[15] und das Totengedenken der Hinterbliebenen, vor allem in Form seiner religiösen Ausprägung (Art. 2 Abs. 1 GG, ggf. Art. 4 Abs. 1 GG)[16], berühren.

### *2. Materielle und formelle Eingriffsvoraussetzungen*

Die Anordnung der Obduktion nach § 25 Abs. 4 S. 2 IfSG ist stets in Zusammenhang mit den allgemeinen Vorgaben zu lesen, die § 25 Abs. 1 IfSG für Ermittlungsbefugnisse aufstellt. Nach seinem ersten Satz stellt das Gesundheitsamt die erforderlichen Ermittlungen an, insbesondere über Art, Ursache, Ansteckungsquelle und Ausbreitung der Krankheit, wenn sich ergibt oder anzunehmen ist, dass ein Verstorbener krank, krankheitsverdächtig oder Ausscheider war.

Nach diesen Vorschriften muss der Verstorbene einer der in § 25 Abs. 1 S. 1 Halbs. 2 IfSG genannten Störergruppen zuzuordnen sein. Es muss jedenfalls anzunehmen sein[17], dass er Kranker, Krankheitsverdächtigter oder Ausscheider i. S. des § 2 Nr. 4–6 IfSG gewesen ist. In professioneller Einschätzung muss das zuständige Gesundheitsamt nach Abs. 4 S. 2 die mit der Obduktion ermöglichten Ermittlungen darüber hinaus für erforderlich halten. Dies bezieht sich *de lege lata* auf den in § 1 Abs. 1 IfSG und im 4. Abschnitt des Gesetzes benannten Zweck, übertragbare Krankheiten beim Menschen „zu bekämpfen“[18]. Konkret muss die Ermittlung durch Obduktion der Gewinnung notwendiger epidemiologischer Erkenntnisse dienen, die § 25 Abs. 1 S. 1 IfSG insbesondere in „Art, Ursache, Ansteckungsquelle und Ausbreitung der Krankheit“ erblickt[19]. Über den Wortlaut hinaus müssen zudem alle weiteren Aspekte des Verhältnismäßigkeitsgrundsatzes geachtet werden[20].

Zur näheren Austarierung herrscht in der Literatur bis heute der – hier zu erörternde (s. III.) – Standpunkt vor, dass eine verhältnismäßig angeordnete Obduktion nach § 25 IfSG einen Zusammenhang zu einer konkreten Schutzmaßnahme nach den – in den letzten zwei Jahren vielfach novellierten – §§ 28 ff. IfSG voraussetze[21]. Die Gesetzesbegründung von 1979 zur Neufassung des § 31 Abs. 1 BSeuchG[22] bedinge, dass der Rückgriff auf die Ermittlungsgeneralklausel, die nun in § 25 Abs. 1 IfSG liegt, dem Zweck dienen müsse, die einzelnen Bekämpfungsmaßnahmen auf eine ausreichende Tatsachenkenntnis zu stützen[23]. „Zweck der Ermittlungen [sei es] festzustellen, ob und ggf. welche Maßnahmen zur Bekämpfung übertragbarer Krankheiten […] getroffen werden müssen.“[24] Aufgrund des gleichgebliebenen Wortlauts des § 25 IfSG wird diese Begründung und die aus ihr resultierende Position bis heute zur Auslegung des § 25 Abs. 1, Abs. 4 S. 2 IfSG herangezogen. Auch die Sektionsanordnung könne nur erforderlich sein, wenn sie der Gewinnung von Erkenntnissen diene, die notwendig sind, um in einem betroffenen Einzelfall faktenbasiert über die Anordnung von erforderlichen Schutzmaßnahmen nach den §§ 28 ff. IfSG entscheiden zu können[25]. Weitergehende Erkenntnismehrungen seien abstrakte wissenschaftliche Forschungen über den Infektionsschutz hinaus, die auch in den Augen des Gesetzgebers keinen legitimen Sektionszweck ausmachten[26].

Die Anordnungszuständigkeit obliegt bei alledem nicht ohne weiteres dem Gesundheitsamt. § 25 Abs. 4 S. 2 IfSG stellt auf eine „zuständige Behörde“ ab und verweist damit über § 54 IfSG auf die nach Landesrecht für zuständig zu erklärende Stelle[27]. Hierdurch können die Länder gestalten, inwieweit das prüfende Gesundheitsamt und die Anordnungsbehörde auseinanderfallen[28].

### *3. Evolution der Norm*

Bereits zu Beginn der SARS-CoV-2-Pandemie hat sich die Legislative der Amtsobduktion nach dem IfSG angenom-

10) Ausführlich zur Unterteilung der Sektionen *Pollähne*, in: *Wienke/Rothschild/Janke*, Rechtsfragen der Obduktion und postmortalen Gewebespende, 2012“ S. 33, 44.
11) Zur Anordnung einer hoheitlichen Sektion (hier nach § 87 StPO) gegen den Willen des Verstorbenen s. BVerfG, NJW 1994, 783; NJW 1994, 783, 784; LG Mainz, NStZ-RR 2002, 43.
12) *Kießling*, in: *Kießling*, IfSG, 3. Aufl. 2022, § 25, Rdnr. 26; *Gabriel*, in: *Eckart/Winkelmüller*, BeckOK IfSG, Stand 1. 7. 2022, § 25, Rdnr. 61.
13) Das postmortale Persönlichkeitsrecht ist in der ständigen Rspr. des BVerfG anerkannt, s. grundlegend BVerfGE 30, 173, 194 („Mephisto“). Die hL leitet das Recht aus Art. 2 Abs. 1 i. V. mit Art. 1 Abs. 1 GG ab, vgl. *Herdegen*, in: *Dürig/Herzog/Scholz*, GG, 97. EL Januar 2022, Art. 1 Abs. 1, Rdnr. 57; *Dreier*, in: *Dreier*, GG, 3. Aufl. 2013, Art. 1 Abs. 1, Rdnr. 76; ebenso *Kunig/Kämmerer*, in: *v. Münch/Kunig*, GG, 7. Aufl. 2021, Art. 2, Rdnr. 85. Die Rspr. sieht hingegen nur eine Verankerung in Art. 1 Abs. 1 GG, exemplarisch BVerfG, NVwZ 2008, 549 f.
14) Zu der Annahme, die Religionsfreiheit wirke postmortal fort, *zu Hohenlohe*, GewArch 2018, 169, 171; *Stern/Sachs/Dietlein,* in: *Stern*, Staatsrecht, Bd. IV/2, 1. Aufl. 2011, § 118 V. 3; *Tag*, in: FS f. *Laufs*, 2006, S. 1079, 1100.
15) BVerfG, NJW 1994, 783; NJW 1994, 783, 784. Erneut ist die Herleitung umstritten: Für eine Herleitung aus Art. 2 Abs. 1 GG, vgl. *Dettmeyer*, Die verfassungsrechtlichen Grenzen für die Einführung einer Verwaltungssektion bei medizinisch unklaren Todesfällen, 1999, S. 184; *Enders*, in: *Merten/Papier*, Handbuch der Grundrechte, Bd. IV, 2011, § 89, Rdnr. 79; HessStGH, NJW 1968, 1923; VG Karlsruhe, NVwZ-RR 2006, 297, 299; für eine Ableitung aus Art. 6 Abs. 1 GG *Schmidt-Didczuhn,* ZRP 1991, 264, 266; *zu Hohenlohe*, GewArch 2018, 169, 173.
16) BVerfG, ZfBR 2016, 582, Rdnr. 62; *zu Hohenlohe*, GewArch 2018, 169, 174; *Schliephorst*, in: *Wienke/Rothschild/Janke*, Rechtsfragen der Obduktion und postmortalen Gewebespende, 2012, S. 23, 24.
17) Eine konkrete Gefahr bzw. ein konkreter Gefahrenverdacht genügt, ThürOVG, BeckRS 2020, 8272, Rdnr. 25; dazu ausführlich *Gabriel*, in: *Eckart/Winkelmüller*, BeckOK IfSG, Stand 1. 7. 2022, § 25, Rdnrn. 10 f.
18) Zur entsprechenden Einordnung als Gefahrenabwehrrecht BVerwGE 142, 205, 216; HmbOVG, COVuR 2020, 105, Rdnr. 11; *Sodan,* in: *Ehlers/Fehling/Pünder*, Besonderes Verwaltungsrecht, 4. Aufl. 2020, § 56, Rdnr. 2; *Mers*, Infektionsschutz im liberalen Rechtsstaat, S. 26 ff.; *Steinau-Steinrueck*, Die staatliche Verhütung und Bekämpfung von Infektionskrankheiten, 2013, S. 91.
19) *Gabriel*, in: *Eckart/Winkelmüller*, BeckOK IfSG, Stand 1. 7. 2022, § 25, Rdnr. 61; vgl. auch BT-Dr. 8/2468, S. 26 zu § 31 BSeuchG.
20) *Gabriel*, in: *Eckart/Winkelmüller*, BeckOK IfSG, Stand 1. 7. 2022, § 25, Rdnrn. 61, 66; *Poscher*, in: *Huster/Kingreen*, Hdb. InfSR, 2. Aufl. 2022, Kap. 4, Rdnr. 71.
21) *Kießling*, in: *Kießling*, IfSG, 3. Aufl. 2022, § 25, Rdnr. 26, § 28, Rdnr. 12; *Erdle*, IfSG, 7. Aufl. 2020, S. 100.
22) Viertes Gesetz zur Änderung des BSeuchG v. 18. 12. 1979, BGBl. I S. 2248.
23) BT-Dr. 8/2468, S. 26; zum hergestellten Konnex etwa *Gerhardt*, IfSG, 6. Aufl. 2022, Vorb. §§ 24 ff., Rdnr. 1.
24) BT-Dr. 8/2468, S. 26.
25) *Madea/Püschel/Lignitz/Dettmeyer*, Rechtsmedizin 2006, 16, 13, 14; *Erdle*, IfSG, 7. Aufl. 2020, S. 100; wohl im Ergebnis ebenso *Gerhardt*, IfSG, 6. Aufl. 2022, Vorb. §§ 24 ff., Rdnr. 1.
26) Vgl. BT-Dr. 8/2468, S. 26 mit einem konstitutiven Bezug aller Ermittlungen zur Bekämpfung übertragbarer Krankheiten, *Gabriel*, in: *Eckart/Winkelmüller*, BeckOK IfSG, Stand 1. 7. 2022 § 25, Rdnr. 61; anknüpfend *Kießling*, in: *Kießling*, IfSG, 3. Aufl. 2022, § 25, Rdnr. 26.
27) Näher *Diesterhöft/Sinder*, in: *Kießling*, IfSG, 3. Aufl. 2022, § 54, Rdnrn. 2 ff.
28) Z. B. ist in NRW das Gesundheitsamt regelmäßig nicht zugleich die landesrechtlich bestimmte „zuständige Behörde“. Gemäß § 3 Abs. 1 IfSBG NRW sind außerhalb kreisfreier Städte die Städte und Gemeinden und damit die jeweiligen örtlichen Ordnungsbehörden „zuständige Behörden“, vgl. *Kießling*, in: *Kießling*, IfSG, 3. Aufl. 2022, § 25, Rdnr. 26.

men, indem der Gesetzgeber das ursprünglich bestehende Entschließungsermessen der zuständigen (Anordnungs-)Behörde reduzierte. Mit Wirkung zum 23.5.2020 änderte er §25 Abs. 4 S. 2 IfSG mit dem *2. COVIfSGAnpG* von einer „Kann"- zu einer „Soll-Vorschrift"[29]. Seitdem muss die zuständige Behörde die Anordnung treffen, wenn das Gesundheitsamt diese für erforderlich hält und der im Einzelfall auch verhältnismäßigen Obduktion keine atypischen Gründe entgegenstehen[30]. Gleichwohl blieb der Beurteilungsspielraum des Gesundheitsamtes hinsichtlich der Erforderlichkeit der Obduktion bestehen („wenn dies […] für erforderlich gehalten wird"). Dennoch wurde bereits mit dieser Novelle greifbar, dass der Gesetzgeber die Bedeutung der Obduktion für den Infektionsschutz erkannte und stärkte. Er begründete die Einführung der „Soll-Vorschrift" damit, dass „die Erkenntnisse aus einer inneren Leichenschau […] insbesondere wertvolle Hinweise auf die Ausbreitung einer Krankheit"[31] liefern könnten. Mit seiner Neuregelung setzte er einen Impuls, das Erkenntnisinstrument der Amtsobduktion im Rahmen der akuten Pandemie tatsächlich zu nutzen.

Mit dem *2. IfSGuaÄndG* trat zum 1.6.2021[32] eine weitere Änderung des §25 IfSG in Kraft, welche die Amtsobduktion[33] befördern sollte. In Reaktion auf die rekordverdächtige Entwicklung von Schutzimpfungen wurde in §25 Abs. 1 S. 2 IfSG festgelegt, dass das Gesundheitsamt die nach §25 IfSG zulässigen Ermittlungen auch anstellen kann, „wenn sich ergibt oder anzunehmen ist, dass jemand durch eine Schutzimpfung oder andere Maßnahme der spezifischen Prophylaxe eine gesundheitliche Schädigung erlitten hat."

Diese Änderung erweiterte den Störerkreis für Obduktionsanordnungen erheblich. Neben den in §25 Abs. 1 S. 1 IfSG genannten Personen können nunmehr auch Obduktionen an Verstorbenen durchgeführt werden, deren Ableben im zeitlichen Zusammenhang mit einer Schutzimpfung oder der Verabreichung von Medikamenten, welche unmittelbar die Viruslast im Körper verringern und damit Schutz vor einer Weiterverbreitung generieren, eingetreten ist[34]. Zugleich markiert diese Regelung – im Lichte der vorherrschenden Interpretation des §25 Abs. 4 S. 2 IfSG (s. I. 2.) – einen bemerkenswerten gesetzessystematischen Bruch: Der Gesetzgeber betritt das Feld der Schutzimpfung und damit der Prophylaxe, die – ebenso wie die zur Therapie eingesetzten Medikamente – kaum noch als eine von den §§28ff. IfSG vorgesehene Schutzmaßnahme erachtet werden kann.

## II. DEFEAT PANDEMIcs – Eindrücke aus der Rechtsanwendung

Zu welcher Praxis führt nun diese rechtliche Ausgangslage? Um diese Frage beantworten zu können und die deutschlandweite Praktikabilität des sog. Hamburger Weges zu ergründen, wurde im Rahmen des NUM eine Pilotstudie aufgelegt. Unter dem Titel DEFEAT PANDEMIcs sollte die Studie die von Amts wegen angeordneten pandemiebezogenen Obduktionen in Deutschland erheben und etwaige Hindernisse für eine deutschlandweite *pandemic preparedness* des Einsatzes von §25 Abs. 4 S. 2 IfSG sowie Verbesserungsvorschläge herausarbeiten, um das möglicherweise brachliegende Potential der Norm aufzudecken[35]. Hierbei kam früh die Hypothese auf, dass Unsicherheiten bei der Rechtsanwendung einem funktionierenden Sektionswesen entgegenstehen.

### *1. Anwendungshäufigkeit*

Die empirische Basis wurde in zwei Schritten erhoben. Zunächst sollte eine Umfrage an die ca. 375 deutschen Gesundheitsämter bestimmen, wie viele pandemiebezogene Obduktionen im Jahr 2020 durchgeführt worden sind und auf welcher Rechtsgrundlage diese beruhten. Der elektronische Survey konnte in 13 mitwirkende Bundesländer verschickt werden. Die pandemiebedingt konstant hohe Arbeitsbelastung der Gesundheitsämter und schwer identifizierbare konkrete Ansprechpartner führten zu einer Rücklaufquote von 41,6%. Von den immerhin 124 rückmeldenden Ämtern hatten 14 (17,36%) überhaupt an einer Obduktionsanordnung mitgewirkt. Für das Jahr 2020 wurde insgesamt nur von 88 Anordnungen berichtet, wovon allein 71 auf Hamburg entfielen. Lediglich ein weiteres Cluster von etwa 10 Anordnungen konnte in Baden-Württemberg festgestellt werden. Außerhalb Hamburgs beruhten die meisten durchgeführten Obduktionen nicht auf §25 Abs. 4 S. 2 IfSG, sondern auf der Zustimmung der Angehörigen im sonst üblichen klinisch-pathologischen Setting.

Trotz der absolut betrachtet geringen Beteiligungsquote konnte die Umfrage ein unter den Bundesländern stark divergierendes Anordnungsverhalten offenbaren. Rechnet man ein, dass fehlende Ansprechpartner für das Thema Obduktion auf eine mangelnde Anwendungspraxis hindeuten, ist jenseits von Hamburg und Baden-Württemberg von einer sehr geringen bis nicht existenten Anwendungshäufigkeit auszugehen.

### *2. Hinderungsgründe: Eindrücke aus der Länderpraxis*

Die innerhalb der ersten Umfrage aufscheinende Anwendungsdivergenz und -abstinenz vertiefte den Bedarf, in einem zweiten Schritt durch eine Serie von Experteninterviews die Gründe für die insgesamt zahlenmäßig geringe Anwendungshäufigkeit zu erforschen. Die qualitativen, einstündigen Interviews wurden mit 17 rechtlichen sowie medizinischen Experten und Expertinnen der oberen Gesundheitsbehörden in sechs verschiedenen Bundesländern sowie des Bundesministeriums für Gesundheit (BMG) geführt[36].

Trotz des stets gesehenen grundrechtlichen Kontexts ließ sich keine prinzipiell ablehnende Haltung gegenüber Amtsobduktionen zugunsten des Infektionsschutzes feststellen; die Obduktion wurde als bedeutsames Instrument bewertet. Eine anfängliche Ansteckungsangst wurde trotz der eingangs Obduktionen ablehnenden Haltung des Robert Koch-Instituts (RKI) nur selten für die Anfangszeit der Pandemie als Hindernis erwähnt. Ersichtlich wurde jedoch, dass der Obduktion als Erkenntnisinstrument im Rahmen der allgemeinen Überlastung der Gesundheitsämter während des gesamten Pandemieverlaufs „regelmäßig keine Priorität" zuteilwurde. Sie war offenbar ein Randthema, wodurch §25 Abs. 4 S. 2 IfSG außerhalb Hamburgs nicht den Rang eines vertrauten und mit Expertise angewandten „Arbeitsparagraphen" erlangte.

Auch die pandemiebedingte Evolution der Norm, die eine Ausweitung der Amtsobduktion fördern sollte, blieb regelmäßig folgenlos. Vor allem die Modifizierung zur „Soll-Vorschrift" im Mai 2020 war in vielen Bundesländern offenbar unbekannt. Allein eine beginnende, aber sporadische Anordnung von Obduktionen hinsichtlich der Implikationen von Schutzimpfungen wurde erkennbar[37].

29) Art. 1 des 2. COVIfSGAnpG v. 19.5.2020, BGBl. I S. 1018 (Nr. 23).
30) Zur Einordnung als intendiertes Ermessen etwa *Gabriel*, in: *Eckart/Winkelmüller*, BeckOK IfSG, Stand 1.7.2022, §25, Rdnr. 65.
31) BT-Dr. 19/18967, S. 59.
32) Art. 1 des IfSGuaÄndG v. 28.5.2021, BGBl. I S. 1174 (Nr. 26).
33) Die Begründung bezieht Abs. 4 explizit mit ein, BT-Dr. 19/29870, S. 33.
34) BT-Dr. 19/29870, S. 33.
35) Das Forschungsprojekt wurde in dem hier betroffenen Arbeitspaket federführend vom Institut für Rechtsmedizin des UKE (Prof. Dr. med. *Klaus Püschel*; PD Dr. med. *Axel Heinemann*; *Hanna-Lisa Goebels*) in Verbindung mit dem Institut für Medizinrecht der Bucerius Law School geleitet.
36) Beteiligt waren Baden-Württemberg, Bayern, Bremen, Hamburg, NRW sowie Sachsen.
37) Nach wie vor scheint in der Praxis indes die vertrautere Anordnung nach den §§87ff. StPO vorzuherrschen, obgleich dies infolge ihres allein strafprozessualen Zwecks sehr zweifelhaft erscheint.

Soweit § 25 Abs. 4 S. 2 IfSG in Hamburg und zum Teil in Baden-Württemberg zur Anwendung gelangte, wurde die Erforderlichkeit weitgehend mit dem Verweis auf das prinzipiell geringe Wissen über den Erreger vor dem Hintergrund der einschneidenden Pandemie bejaht. Eine entsprechend weite Deutung wurde in Hamburg durch eine Einschätzung aus der für Rechtsfragen des IfSG zuständigen Abteilung des BMG „abgesichert", die anderen Bundesländern wiederum unbekannt blieb.

Fernab der beiden genannten Bundesländer zeichnete sich im Rahmen der erbetenen Bewertung des § 25 Abs. 4 S. 2 IfSG der Tenor ab, dass die Norm insbesondere hinsichtlich der zu prüfenden Erforderlichkeit und damit auch in ihrer Reichweite unklar sei, weshalb ihre Meidung durch die Praxis nicht verwundern könne. Sie erschöpfe sich in einem wenig hilfreichen Appell, die Verhältnismäßigkeit zu wahren. In diesem Licht sind auch – letztlich ausweichende – Kooperationsmodelle mit pathologischen Instituten zu sehen, mit denen klinische Obduktionen auf der Basis von Zustimmungslösungen forciert worden sind, um im Kontext der Pandemie Erkenntnisse zu mehren. Zum Teil wurde die Norm unter Rückgriff auf die referierten Kommentierungen im Zuge der SARS-CoV-2-Pandemie explizit als faktisch unanwendbar bewertet. Mit dem Argument, Obduktionen wiesen bei der Anordnung in Kohorten keinen konkreten Ermittlungsbezug zu einer einzelnen, durch Schutzmaßnahmen zu durchbrechenden Infektionskette mehr auf und würden allein einen „kurativen Zweckgewinn" verfolgen, wurde der Rückgriff auf § 25 Abs. 4 S. 2 IfSG abgelehnt. Lediglich bei Ausnahmefällen mit Verdachtsmomenten neuer Erregertypen wurde eine Anordnung erwogen.

Neben den Unsicherheiten hinsichtlich der Deutung der Erforderlichkeit wurde das Anordnungsprozedere kritisiert und auch dahingehend eine Verbesserung der überaus „schlanken" rechtlichen Grundlagen erbeten. Gerade im Vergleich zu landesrechtlichen Sektionen seien formelle Verfahrensschritte und Folgefragen unklar. Vor allem die Beteiligung der Angehörigen sei ohne klare Bestimmungen herausfordernd. Erneut wurde eine restriktive Normhandhabung erkennbar, indem bei der Amtsobduktion zum Teil Widersprüche beachtet und mehrheitlich Zustimmungen zur Durchführung angestrebt wurden. Auch im Rahmen des sog. Hamburger Wegs wurde jeweils versucht, Zustimmungserklärungen einzuholen.

## III. Resultierende Hauptfrage – die Reichweite des geltenden Rechts

Diese Zahlen und plausiblen Anwendungsbeobachtungen legen nahe, dass ein im Kern zufälliges, in Hamburg offenbar vorhandenes und zu Nachfragen bis hin zum BMG führendes Erkenntnisinteresse zu dem heute als vorbildhaft geltenden „Hamburger Weg" geführt hat. Im Übrigen wird sich sagen lassen, dass § 25 Abs. 4 S. 2 IfSG kein „Arbeitsparagraph" ist, sondern eine kaum bekannte und *in praxi* gemiedene Regelung darstellt. Auch in einer einschneidenden Pandemie fiel die Amtsobduktion deshalb aus; allenfalls mutierte sie außerhalb Hamburgs zu einer konsensual in Einzelfällen durchgeführten Obduktion[38], für die in Deutschland ohnedies immer geringere Quoten berichtet werden[39].

Diese Praxis, die eine sog. *pandemic preparedness* nicht vermuten lässt, könnte jedoch ihre Berechtigung haben, wenn die rechtlichen Bedenken, welche eine breitere Anwendung neben der Überlastung der Gesundheitsämter offenbar hinderten, überzeugen. Nicht die ausbleibende Praxis, sondern der „Hamburger Weg" könnte – rechtlich betrachtet – das Skandalon sein. Deshalb ist näher zu klären, ob und inwiefern § 25 Abs. 4 S. 2 IfSG ein legitimes Mittel ist, um in neu auftretenden Pandemien Erkenntnisse zu mehren – seine Reichweite ist zu bestimmen.

### *1. Systematik: Zustimmungsbedarf bei allgemeiner Forschung*

Wie bereits unter I. 2. vermerkt wurde, besteht in der Literatur Einigkeit, dass die Anordnung nach § 25 Abs. 4 S. 2 IfSG *nicht ausschließlich* einem wissenschaftlichen Interesse dienen darf[40]. Diese Annahme speist sich aus der zutreffenden Auffassung[41], dass die Leichenöffnung ohne Zustimmung sowohl die Hinterbliebenenrechte der Angehörigen als auch den postmortalen Grundrechtsschutz des Verstorbenen tangiert[42]. Jedenfalls bei einer fehlenden Zustimmung muss ein gesetzlich normierter Sektionszweck die Anordnung der Obduktion zu rechtfertigen vermögen[43], indem der gesetzlich verfolgte legitime Zweck die Grundrechtsbeeinträchtigungen einzelner Betroffener überwiegt[44]. Diese Zwecke werden zum Teil bereits in der Qualitätssicherung und -kontrolle des Gesundheitswesens oder der Erstellung einer genauen Mortalitätsstatistik erkannt, da diese den medizinischen Fortschritt in Form der Identifikation neuer Erkrankungen oder der verbesserten Grundlagenforschung für neue Therapieansätze fördern könnten[45]. Dem ist jedoch in dieser Abstraktheit nicht zu folgen[46], weil insbesondere der postmortale Achtungsanspruch als Ausdruck grundrechtlicher Freiheit im Umgang mit der Leiche anderenfalls per se geleugnet wäre: Die abstrakt stets bestehende Notwendigkeit medizinischen Fortschritts würde die Leiche der subjektiven Verfügungsfreiheit des Grundrechtsträgers entziehen und dem gesellschaftlichen Bedürfnis überantworten; seine individuelle Haltung zur Forschung und zur Bedeutung des Restbestandes seiner Existenz wäre irrelevant[47], obschon gerade die Einordnung in Art. 2 Abs. 1 i. V.m 1 Abs. 1 GG[48] einen grundsätzlich

38) Diese Annahme bestätigend *Boor et al.*, Pathologe, 2021, 40, 197, 200.

39) Die Zahl gerichtlich veranlasster Obduktionen rangiert stabil bei rund 2 % der über 800.000 Todesfälle im Jahr, *Madea/Rothschild*, DÄBl., 2010, 107, 575, 585; *Schröder/Püschel*, Bundesgesundheitsbl., 2019, 62, 1438, 1442. Die Rate klinischer Obduktionen wurde im Jahr 2014 bei Unikliniken mit 8,6 % und bei kommunalen Krankenhäusern mit 3,8 % aller Todesfälle in Krankenhäusern angegeben, wobei die absolute Zahl durchgeführter Obduktionen Untersuchungen zur Folge zwischen den Jahren 2005 und 2014 um 30 % zurückgegangen ist, *Grassow-Narlik et. al.*, Pathologe, 2017, 38, 422, 426. Nach Angaben führender Rechtsmediziner reduzierte sich die Quote klinischer Obduktionen im Jahr 2017 weiter auf 1–3 % der Todesfälle in Krankenhäusern.

40) Vgl. bereits Fn. 26.

41) *Gabriel*, in: *Eckart/Winkelmüller*, BeckOK IfSG, Stand 1. 7. 2022, § 25, Rdnr. 61; *Gerhardt*, IfSG, 6. Aufl. 2022, § 25, Rdnr. 54; *Lipp*, in: *Laufs/Katzenmeier/Lipp*, Arztrecht, 8. Aufl. 2021, VI, Rdnr. 84.

42) Zu den konkret beeinträchtigten Grundrechten s. bereits Fn. 13–16.

43) *Lipp*, in: *Laufs/Katzenmeier/Lipp*, Arztrecht, 8. Aufl. 2021, VI, Rdnrn. 84 f.; ausführlich zu den Legitimationsgrundlagen der Sektion *Duttge,* in: *Groß/Esser/Knoblauch/Tag*, Tod und toter Körper, 2007, S. 117, 120 ff.; *Pollähne*, in: *Wienke/Rothschild/Janke*, Rechtsfragen der Obduktion und postmortalen Gewebespende, 2012, S. 33, 44.

44) Vgl. zum Abwägungsprozess bei der Gerichtssektion, die beiden Entscheidungen, BVerfG, NJW 1994, 783; NJW 1994, 783 f.; unmittelbar zu § 25 Abs. 4 IfSG *Lipp*, in: *Laufs/Katzenmeier/Lipp*, Arztrecht, 8. Aufl. 2021, VI, Rdnr. 85 und *Duttge*, in: *Groß/Esser/Knoblauch/Tag*, Tod und toter Körper, 2007, S. 117, 121.

45) Vgl. *Franzki*, MedR 1991, 223, 225 f.

46) I. E. wie hier etwa schon *Lipp*, in: *Laufs/Katzenmeier/Lipp*, Arztrecht, 8. Aufl. 2021, VI, Rdnr. 84; *Duttge*, in: *Groß/Esser/Knoblauch/Tag*, Tod und toter Körper, 2007, S. 117, 122. A. A. *Böckle*, Pathologe 983, 4, 1 f., der verkennt, dass der Achtungsanspruch mit der die menschliche Würde begründenden Selbstbestimmung verknüpft ist.

47) In der Sache bereits *Duttge*, in: *Groß/Esser/Knoblauch/Tag*, Tod und toter Körper, 2007, S. 117, 122.

48) S. zur dogmatischen Herleitung des postmortalen Persönlichkeitsschutzes Fn. 13.

starken Grundrechtsschutz bedingt[49]. Selbst wenn man von einem oft geteilten Interesse des Verstorbenen ausgeht, sein Ableben medizinisch gewinnbringend für die Menschheit auszuwerten[50], kann dieses Interesse nicht für jedermann gesetzlich fingiert werden, zumal die empirische Realität der klinischen Sektion eine gegenteilige Einschätzung bei den betroffenen Menschen nennenswert in den Raum stellt. Soll die subjektive Rechtsposition ernsthaft geachtet sein, muss eine Zwangsobduktion vielmehr auf einem einzelfallbezogenen hinreichend gewichtigen medizinischen oder gesellschaftlichen Erkenntnisinteresse beruhen, das ausnahmsweise eine partielle Zurücksetzung des ggf. von den Angehörigen durchgesetzten Achtungsanspruchs gestattet[51]. Überdies wäre ein allgemeiner Rekurs auf die Wissenschaftsfreiheit (Art. 5 Abs. 3 S. 1 GG) unbehilflich, da diese den Forschenden keine Eingriffsrechte bezüglich der Rechte anderer Grundrechtsträger gewährt[52] – die Hinwendung zur Wissenschaft bleibt vielmehr der höchstpersönlichen Entscheidung der Betroffenen anvertraut.

Anderes kann zudem nicht zugrunde gelegt werden, weil der 5. Abschnitt des IfSG, in den sich § 25 IfSG einfügt, nur Ermittlungen zur „Bekämpfung einer Krankheit" betrifft. Die Obduktion zugunsten einer hiervon nicht mehr motivierten Grundlagenforschung wird schon nicht mehr von der Regelung getragen, die der Gesetzgeber tatsächlich geschaffen hat. Die Forderung nach einem die zwangsweise Anordnung legitimierenden Grund, welcher hier in epidemiologischen Erwägungen zur Bekämpfung übertragbarer Krankheiten liegen muss, ist daher überzeugend.

### *2. Bedeutung der Gefahrenabwehr nach dem IfSG: Schutz über konkrete Infektionsketten hinaus*

Diese Bestätigung der allgemeinen Konturen des Auslegungsstandes beantwortet jedoch die eigentliche Frage nicht, welche epidemiologischen Erkenntnisgewinne mit der Obduktion konkret gefördert werden dürfen, also i. S. d. IfSG erforderlich sind. Zu klären ist, ob tatsächlich – wozu die Länderpraxis anhand der an konkreten Einzelmaßnahmen modellierten Kommentarliteratur tendiert – ein angestrebter Erkenntnisgewinn zu fordern ist, mit dem über Schutzmaßnahmen zur Durchbrechung einzelfallbezogener Infektionsketten nach den §§ 28 ff. IfSG befunden werden kann oder ob – wie mit dem „Hamburger Weg" angenommen – eine umfassendere, der Bekämpfung einer Infektionskrankheit dienliche Wissensmehrung legitim ist[53].

#### a) Historische Perspektive

Die tendenziell engführenden Stellungnahmen[54] berufen sich auf einen historischen Gesetzgeberwillen, der bei der Neufassung von § 31 BSeuchG ein Bedingungsverhältnis zwischen Ermittlungs- und Schutzmaßnahmen angestrebt habe (vgl. bereits I.2.). Jedoch ergibt sich daraus nicht die Intention, Ermittlungen nur in Fällen zu gestatten, in denen Anordnungen zur Durchbrechung einzelner konkreter Infektionsketten in Rede stehen. Die Gesamtschau der damaligen Gesetzesbegründung zeigt vielmehr, dass der Gesetzgeber 1979 primär anstrebte, Ermittlungsmaßnahmen bereits in der Verdachtsphase einer Infektion zu gestatten, indem die Tatbestandsvariante „ist anzunehmen" geschaffen wurde, welche früher ansetzende und damit effizientere Schutzmaßnahmen ermöglicht[55]. Die Aufklärung einer bestehenden Infektionskrankheit sollte sichergestellt, nicht aber die Bindung an die Durchbrechung einer konkreten Infektionskette hergestellt werden. Auch der Wortlaut von § 25 IfSG spiegelt diese Forderung nach einer so eng verstandenen Verflechtung nicht wider.

Ferner verbürgt der Umstand nichts anderes, dass im Jahr 2000 eine über den Bundesrat geforderte Präzisierung einer Vorgängernorm verworfen wurde, nach der eine Obduktion „zur Bekämpfung übertragbarer Krankheiten oder um wichtige infektionsepidemiologische Erkenntnisse im Interesse verbesserter Verhütungs- und Bekämpfungsmaßnahmen"[56] gewinnen zu können, zulässig sein sollte. Über die Ratio des abgelehnten Antrags ist lediglich die Stellungnahme der damaligen Bundesregierung bekannt, nach der „die Regelungen zur inneren Leichenschau" bereits „ausreichend" seien[57]. Diese Begründung lässt Rückschlüsse zu, dass das vom Bundesrat angestrebte Anwendungsspektrum vom damaligen § 26 Abs. 3 S. 2 IfSG a. F. bereits umfasst sein sollte. Eine eindeutige Stellungnahme zu der Frage, ob ein Konnex zu einer einzelnen Infektionskette bestehen müsse, ist der Gesetzgebung jedenfalls nicht zu entnehmen.

#### b) Horizont der SARS-CoV-2-Pandemie

Interpretiert man die §§ 25 ff. IfSG jedoch auf dem Erkenntnis- und (zwischenzeitlichen) Rechtsstand der SARS-CoV-2-Pandemie, vermag allein der weitere Auslegungsansatz zu überzeugen:

Obduktionen, die der gezielten Ermittlung der notwendigen lokalen Einzelmaßnahmen dienen und damit einzelne Infektionsketten eindämmen sollen, sind zwar Musterbeispiele für zulässige Anordnungen. Die SARS-CoV-2-Pandemie hat aber gezeigt, dass allein lokale Schutzmaßnahmen bei einer raumgreifenden Verbreitung eines hochansteckenden und globalen Erregers nicht ausreichen, um dessen Weiterverbreitung überhaupt nur einzuschränken[58]. Sowohl die Rechtsanwender als auch der im weiteren Verlauf novellierende Gesetzgeber erkannte, dass bei einer akuten Pandemielage, die von einer nennenswerten Unkenntnis über das Virus sowie einer geringen gesellschaftlichen Immunität gekennzeichnet ist, auch großflächige kettenunabhängige Maßnahmen zulässig sein müssen. Die ehedem als Bekämpfungsmaßnahmen vor allem, aber auch nicht ausschließlich[59], tradierten Einzelmaßnahmen, insbe-

49) Bei alledem ist aber zu beachten, dass es sich um eine Nachwirkung des zu Lebzeiten erworbenen Schutzes handelt, s. *Duttge*, in: *Groß/Esser/Knoblauch/Tag*, Tod und toter Körper, 2007, S. 117, 120 f., wenngleich die Einordnung als reine Schutzpflicht angesichts der subjektivrechtlichen Wurzel und der Aktualisierung über die Angehörigen einen Schritt zu weit geht.

50) In diesem Sinne etwa *Tag*, in: FS f. *Laufs*, 2006, S. 1079, 1100: grundsätzlich nachwirkender mutmaßlicher Wille, soweit kein entgegenstehender subjektiver Wille der Betroffenen bekannt sei.

51) So bereits *Duttge*, in: *Groß/Esser/Knoblauch/Tag*, Tod und toter Körper, 2007, S. 117, 122. Vgl. auch § 4 Abs. 2 SektG Hmb, wonach eine klinische Obduktion aus ärztlicher Sicht dringend zur Fürsorge für die Hinterbliebenen oder zur Qualitätssicherung geboten sein muss, um die fehlende Einwilligung der Betroffenen zu überwiegen (Nr. 3).

52) *Britz*, in: *Dreier*, GG, 3. Aufl. 2013, Art. 5 Abs. 3 (Wissenschaft), Rdnrn. 58, 62. Eine Ausnahme kommt lediglich in Frage, wenn Dritte den Wissenschaftsbetrieb unmittelbar angreifen, vgl. *Gärditz*, in: *Dürig/Herzog/Scholz*, GG, 97. EL Januar 2022, Art. 5 Abs. 3, Rdnr. 269.

53) Diese beiden Ansichten bereits in der Literatur gegenüberstellend, *Kießling*, in: *Kießling*, IfSG, 3. Aufl. 2022, § 25, Rdnr. 26.

54) Zu den Vertretern dieser Ableitung s. Fn. 25.

55) Vgl. BT-Dr. 8/2468, S. 26; *Gabriel*, in: *Eckart/Winkelmüller*, BeckOK IfSG, Stand 1. 7. 2022, § 25, Rdnr. 5.

56) BT-Dr. 14/2530, S. 92.

57) BT-Dr. 14/2530, S. 97.

58) Vgl. *Kloepfer*, VerwArch 2021, 169, 184 ff.; *Kießling*, JZ 2022, 53 ff., die weitergehend eine Abkehr des Infektionsschutzrechts von der Gefahrenabwehr hin zu einer planerischen Risikovorsorge fordert.

59) Zum einen zeugte bereits die beispielhafte Aufzählung in § 28 Abs. 1 S. 2 IfSG in der vor März 2020 geltenden Fassung, wonach „Veranstaltungen oder sonstige Ansammlungen einer größeren Anzahl von Menschen beschränk[t] oder [verboten] und Badeanstalten oder in § 33 genannte Gemeinschaftseinrichtungen [..]" geschlossen werden können, dass in Konkretisierung der mit der Generalklausel eröffneten Handlungsmöglichkeiten auch weitreichende Maßnahmen gegenüber der Allgemeinheit bereits vor Ausbruch der Pandemie in Betracht kamen, vgl. *Johann/Gabriel*, sondere des § 28 IfSG a. F.[60], mussten um eine allgemeinere und flächendeckende Dimension weiterentwickelt werden, um die überregionale Virusverbreitung eindämmen zu können[61]. Schon zu Beginn der Pandemie stand insofern eine mit der Verordnungsermächtigung des § 32 IfSG a. F. verknüpfte[62] Generalklausel in § 28 Abs. 1 IfSG zur Verfügung. Sie gestattete jedenfalls im Jahr 2020 eine nicht nur an einzelnen konkreten Infektionsketten ansetzende eingriffsintensive Gefahrenabwehr[63], wurde jedoch im Zuge der von der Rechtsordnung nach und nach klarer begriffenen Dimensionen der freiheitseinschränkenden Folgen einer neuartigen Pandemie zu Recht im November 2020 durch den präzisierend wirkenden § 28a IfSG ergänzt[64]. Speziell zur Verhinderung von COVID-19 konnten bis zur Anpassung des IfSG zum 19. 3. 2022[65] für ganz Deutschland, im Laufe der Zeit auch unabhängig von der Feststellung einer epidemischen Lage von nationaler Tragweite[66], flächendeckende Schutzmaßnahmen, wie u. a. weitgehende Maskenpflichten (§ 28a Abs. 1 Nr. 2 bzw. Abs. 7 Nr. 3 IfSG a. F.), Abstandsgebote im öffentlichen Raum (§ 28a Abs. 1 Nr. 1 bzw. Abs. 7 Nr. 1 IfSG a. F.) sowie vollständige Ausgangs- und Kontaktbeschränkungen im privaten und öffentlichen Raum (§ 28a Abs. 1 Nr. 3 bzw. Abs. 7 Nr. 2 IfSG a. F.) ohne konkreten Bezug zu einer Infektionskette verhängt werden. Auch nach der jüngsten Novelle der Schutzmaßnahmen, die weiterhin unabhängig von einer epidemischen Lage von nationaler Tragweite angeordnet werden können[67], stehen erhebliche einzelkettenüberschreitende Maßnahmen, wie bspw. die Maskenpflicht im ÖPNV (§ 28b Abs. 1 Nr. 1 IfSG), bundesweit zur Verfügung.[68]

In einer Lage, in der Entscheidungen über diesen flächendeckenden Infektionsschutz zu treffen sind, muss nun aber auch die Interpretation der nach § 25 Abs. 1 S. 1 IfSG zu fördernden Erkenntnisgewinne anhand dieser umfassenderen Schutzstrategie erfolgen. Eine Erhebung von breit angelegten, auch ganz grundlegenden Informationen über „Art, Ursache, Ansteckungsquelle und Ausbreitung der Krankheit" bspw. in Form erregertypischer Verbreitungswege oder des letalen Verlaufs der Erkrankung bzw. neuer Mutationen, betrifft sodann nicht „nur" allgemeine Forschung; sie stellt vielmehr einen – wenngleich nicht mit zwingenden Erfolgen versehenen – funktionalen Beitrag zu einer verhältnismäßigen Gefahrenabwehr dar. Indem ein verbesserter Informationsstand die Beurteilung der pandemischen Lage erleichtert, kann er sowohl zum notwendigen Schluss auf unvermeidliche großflächige kettenunabhängige Maßnahmen zur Bekämpfung der Krankheit als auch zur Abstandnahme von nicht (mehr) erforderlichen Grundrechtseingriffen beitragen. Hiermit bestehen jenseits der Wissenschaftsförderung akutere Gründe, die für die Legitimation eines Eingriffs in Grundrechte der Betroffenen in Betracht kommen.

Überdies spricht die Evolution von § 25 IfSG selbst für einen weiteren Anwendungsbereich der Amtsobduktion. Denn mit der Einführung der „Soll-Vorschrift" in § 25 Abs. 1 S. 2 IfSG hat der Gesetzgeber die Obduktion gerade im Angesicht in: *Eckart/Winkelmüller*, BeckOK IfSG, Stand 1. 7. 2022, § 28, Rdnr. 31.1 m. w. N. Zudem hat der Gesetzgeber durch seine Ergänzung von § 28 Abs. 1 S. 1 IfSG um den 2. Halbsatz „sie kann insbesondere Personen verpflichten, den Ort, an dem sie sich befinden, nicht oder nur unter bestimmten Bedingungen zu verlassen oder von ihr bestimmte Orte oder öffentliche Orte nicht oder nur unter bestimmten Bedingungen zu betreten" zum 28. 3. 2020 kenntlich gemacht, dass über punktuell wirkende Maßnahmen hinaus allgemeine oder gleichsam flächendeckende Verbote erlassen werden können, so etwa auch OVG NRW, BeckRS 2020, 6136, Rdnr. 27. Die Verknüpfung mit der Verordnungsermächtigung (§ 32 S. 1 IfSG) ließ ebenfalls bereits vor Pandemiebeginn erkennen, dass es nach Vorstellung des Gesetzgebers auf größerer Fläche eine Vielzahl von Adressaten geben kann, *Kluckert*, in: *Kluckert*, Das neue Infektionsschutzrecht, 2. Aufl. 2021, § 2, Rdnr. 95; OVG NRW, COVuR 2020, 423, Rdnrn. 32 ff.

60) § 28 Abs. 1 S. 1 IfSG in der am 28. 3. 2020 geltenden Fassung, BGBl. I S. 587: „Werden Kranke, Krankheitsverdächtige, Ansteckungsverdächtige oder Ausscheider festgestellt oder ergibt sich, dass ein Verstorbener krank, krankheitsverdächtig oder Ausscheider war, so trifft die zuständige Behörde die notwendigen Schutzmaßnahmen, insbesondere die in den §§ 29 bis 31 genannten, soweit und solange es zur Verhinderung der Verbreitung übertragbarer Krankheiten erforderlich ist; sie kann insbesondere Personen verpflichten, den Ort, an dem sie sich befinden, nicht oder nur unter bestimmten Bedingungen zu verlassen oder von ihr bestimmte Orte oder öffentliche Orte nicht oder nur unter bestimmten Bedingungen zu betreten."

61) S. mit ausführlichen Nachweisen, *Kießling*, JZ 2022, 53, 55 ff. Nachdem in der ersten Pandemiephase Behörden Allgemeinverfügungen auf Grundlagen der Generalsklausel in § 28 Abs. 1 S. 1 IfSG anordneten und die Landesregierungen nach § 28 i. V. mit 32 IfSG Rechtsverordnungen erließen, die u. a. Ausgangssperren, Kontakt-, Reise-, Veranstaltungs- und Versammlungsverbote sowie Schließungen von Bildungs- und Kultureinrichtungen umfassten, mehrten sich die Zweifel an der Verfassungsmäßigkeit dieses Vorgehens. Spätestens ab Herbst 2020 wurde mehrheitlich vertreten, dass die Anordnung von Allgemeinverfügungen und der Erlass von Rechtsverordnungen auf Grundlage von Voraussetzungen einer Generalklausel nicht dem Parlamentsvorbehalt für die damit zu verantwortenden Grundrechtseingriffe genüge, *Kluckert*, in: *Kluckert*, Das neue Infektionsschutzrecht, 2. Aufl. 2021, § 2, Rdnrn. 105 ff. mit umfassenden Nachweisen der diese Ansicht stützenden Judikatur in Fn. 234, s. exemplarisch BayVGH, COVuR 2020, 884, Rdnr. 11; ebenso *Kingreen*, in: *Huster/Kingreen*, Hdb. InfSR, 2. Aufl. 2022, Kap. 1, Rdnrn. 134 f. und *Kießling*, in: *Kießling*, IfSG, 3. Aufl. 2022, § 28, Rdnrn. 54 f. Auch der Gesetzgeber erkannte einen Handlungsbedarf, vgl. BT-Dr. 19/23944, S. 21. Ob bei alledem wegen der nicht mehr konkret personenbezogen notwendigen Gefahrenfeststellung schon von einer Risikovorsorge gesprochen werden sollte, kann hier dahinstehen, weil es in der Sache um die Abwehr einer bereits festgestellten materialisierten Gefahr in der Gesellschaft geht.

62) Zur Bedeutung insbesondere hinsichtlich eines erweiterten Adressatenkreises etwa *Kluckert*, in: *Kluckert*, Das neue Infektionsschutzrecht, 2. Aufl. 2021, § 2, Rdnrn. 91 f., 95; *Rixen*, NJW 2020, 1097, 1101; *Johann/Gabriel*, in: *Eckart/Winkelmüller*, BeckOK IfSG, Stand 1. 7. 2022, § 28, Rdnr. 21; aus der Rspr. HmbOVG, BeckRS 2020, 9944, Rdnr. 21; BayVGH BeckRS 2020, 10399, Rdnr. 36; zum Gesetzgeber BT/Dr. 14/2530, S. 74; a. A. *Kießling*, JZ 2022, 53, 54.

63) Zu dem im Einzelnen differenzierteren Meinungsbild m. w. N. *Kluckert*, in: *Kluckert*, Das neue Infektionsschutzrecht, 2. Aufl. 2021, § 2, Rdnr. 107; *Kießling*, IfSG (1. Aufl.), § 28, Rdnr. 65 mit dem Gebot zu einer restriktiven Auslegung; zusf. VerfGH des Saarlandes, NVwZ 2020, 1513, 1518, Rdnr. 89; weitergehend zulassend OVG NRW, BeckRS 2020, 6136, Rdnrn. 20 ff.; OVG LSA, BeckRS 2020, 8018, Rdnr. 23; HessVGH, BeckRS 2020, 5639, Rdnr. 25; VG Hamburg, BeckRS 2020, 22588, Rdnrn. 33 ff.; *Rixen*, NJW 2020, 1097, 1098 f. (aber mit 1103); *Johann/Gabriel*, in: *Eckart/Winkelmüller*, BeckOK IfSG, Stand 1.7.2022, § 28, Rdnrn. 5, 31.1; *Gerhardt*, IfSG, 6. Aufl. 2022, § 28, Rdnr. 7. Zur Kritik an einer auf Dauer bzw. über eine angemessene Reaktionszeit hinaus aufrechterhaltenen Berufung auf die schmalen §§ 28, 32 IfSG a. F. s. m. w. N. *Kingreen*, in: *Huster/Kingreen*, Hdb. InfSR, 2. Aufl. 2022, Kapitel 1, Rdnrn. 134 ff.; *Kluckert*, in: *Kluckert*, Das neue Infektionsschutzrecht, 2. Aufl. 2021, § 2, Rdnrn. 105 ff.; *Volkmann*, NJW 2020, 3153, Rdnrn. 6, 21; BayVGH, BeckRS 2020, 28521, Rdnrn. 29 f.

64) Art. 1 IfSGMaßAufhG vom 18. 11. 2020, BGBl. I S. 2397, dazu BT-Dr. 19/723944, S. 2. Aber weiter krit. *Kießling*, JZ 2022, 53, 55; *Kingreen*, NJW 2021, 2766, 2768. Zur Rspr. ferner BVerfG, NJW 2022, 139 und 167.

65) Art 1 IfSGMaßAufhG v. 18. 03. 2022, BGBl. I S. 466.

66) Mit Änderung des § 28a Abs. 7 IfSG a. F. durch Art. 1 EpiLageAufhG zum 24. 11. 2021 konnten die flächendeckenden Schutzmaßnahmen in Abs. 1 auch mit Aufhebung der epidemischen Lage von nationaler Tragweite, mit Ausnahme der Ausgangssperren, aufrechterhalten werden, BGBl. I S. 4906.

67) Bei erneuter Feststellung einer epidemischen Lage von nationaler Tragweite steht auch nach der neuesten Novelle weiter das altbekannte umfassende Maßnahmenkonvolut zur Verfügung, § 28a Abs. 1 IfSG.

68) Art. 1a des CovidIfSGAnpG 2022 v. 16. 9. 2022, BGBl. I S. 1454, 1462 ff. (Nr. 32). Die Regelung wird mindestens bis zum 7. 4. 2023 fortdauern.

ihres Einsatzes gegenüber einem noch weithin unbekannten Erreger nicht für einzelne Infektionsketten, sondern vor dem Hintergrund der belastenden flächendeckenden Maßnahmen fördern wollen. Mit Eröffnung der Obduktion für (potentiell) tödliche Impfschäden oder Nebenwirkungen durch § 25 Abs. 1 S. 2 IfSG (I. 3.) darf das Gesundheitsamt nun sogar gegenüber Personen ermitteln, bei denen keine Infektion mit einem Krankheitserreger vermutet bzw. festgestellt wurde, sondern Komplikationen nach Impfstoffapplikation kausal zu bewerten sind. Die Adressaten der Maßnahme stehen nicht einmal länger in einem Gefährdungszusammenhang zu einem konkreten Infektionsherd. Diese auch mit der Erstreckung auf anderweitige Verbreitungsprophylaxen fortgeführte Erweiterung bricht nicht nur mit der früheren Bindung an den Kontext der zwangsweise angeordneten Schutzmaßnahmen. Sie bestätigt, dass der Gesetzgeber von einem breiten Bedarf ausgeht, die Pandemiebekämpfung über die Unterbrechung einzelner konkreter Infektionsketten hinaus durch die Mehrung vorhandener Kenntnisse zu fördern.

### *3. Konkretisierungen*

Die Annahme eines weiten Anwendungsbereichs der Amtsobduktion nach dem IfSG bedarf jedoch der Konkretisierung. Eine epidemiologische Wissensmehrung um jeden Preis, die ohne jede Relation zu den mit ihr ausgelösten Grundrechtseingriffen erfolgt, darf § 25 Abs. 4 S. 2 IfSG nicht auslösen. Nach dem geltenden Verhältnismäßigkeitsgrundsatz ist die Durchführung großangelegter Autopsiekohorten an die aktuelle Entwicklung der epidemischen Lage, an den jeweils erreichten Wissensstand sowie die noch zu erwägenden Bekämpfungsmaßnahmen zu koppeln.

Besonders zu Beginn einer akuten Pandemie ist demnach eine großangelegte und rasche Wissensmehrung auch unter Zwang verhältnismäßig, wenn die bis dato fehlenden Erkenntnisse über ein grassierendes lebensgefährliches Virus verbessert werden müssen, um einer Überlastung des Gesundheitswesens suffizient vorbeugen zu können. Ist die Pandemie hingegen aufgrund bereits gewonnener Erkenntnisse, funktionierender Schutzmaßnahmen oder einer großflächigen gesellschaftlichen Immunität „beherrschbar" (geworden) und insbesondere eine Überlastung des Gesundheitssystems nicht zu befürchten, endet die Rechtfertigung für flächendeckende Zwangsmaßnahmen und mit ihr der Rückgriff auf eingriffsintensive Maßnahmen zur Sachverhaltsermittlung. Die zwangsweise Anordnung setzt stets eine aktuelle, seitens des Gesundheitsamts und der anordnenden Behörde bejahte Dringlichkeit der Informationsgewinnung voraus. Insoweit kann aber auch bei einem bereits besser verstandenen Virus die Verhältnismäßigkeit zu bejahen sein, wenn entdeckte Mutationen ggf. breitere Bekämpfungsmaßnahmen erfordern und diesbezüglich den Erkenntnisbedarf wieder anwachsen lassen.

Darüber hinaus sollte der ggf. von den Hinterbliebenen artikulierte (mutmaßliche) Wille des Verstorbenen bei der Verhältnismäßigkeitsprüfung nicht völlig unberücksichtigt bleiben. Trotz der Einordnung als hoheitliche Sektion sollten die Angehörigen zunächst – wenn möglich auch bei einer akuten Pandemielage – vor Durchführung der Obduktion benachrichtigt und in der Regel angehört werden, damit sie ihren Rechten Ausdruck verleihen und falsche Annahmen ausgeräumt werden können. Aus dem amtlichen Charakter der Sektion sollte nicht vorschnell auf eine verfahrensmäßige Zurücksetzung der Angehörigen geschlossen werden. Inwieweit dann zum Beispiel aufgrund von religiösen Überzeugungen geltend gemachte Widersprüche zu beachten sind, ist wiederum anhand des Pandemieverlaufs und der Gefährdungslage zu bestimmen. Hierbei ist den Akteuren aber ein professioneller Beurteilungsspielraum hinsichtlich des Erkenntnisstandes und der zu erhoffenden Wissensmehrung zuzugestehen, weil anderenfalls die Sorge vor *ex post* gemachten Beschwerden die Amtsobduktion wie bisher lähmen dürfte.

Zu Beginn einer neuartigen Pandemie, wenn jede Obduktion noch regelmäßig einen erheblichen Beitrag zum Wissensstand leisten kann, wird sich die zwangsweise Anordnung gemäß § 25 Abs. 4 S. 2 IfSG demnach legitimieren lassen. Zu diesem Zeitpunkt müssen mögliche Widersprüche hinter gesamtgesellschaftlichen Erwägungen des Freiheit- und Gesundheitsschutzes zurücktreten. Dies gilt insbesondere, wenn die Obduktion in eine nationale Erkenntnissammlung eingebunden ist und damit eine höhere Signifikanz der Forschungsergebnisse erzielt werden kann. Umso mehr sich Forschungsansätze jedoch bereits erhärten oder zu verwerfen sind – welches zugleich die Grenze des Beurteilungsspielraums der Akteure konstituiert – desto eher werden Erkenntnisse aus einzelnen Obduktionen verzichtbar sein. Jedenfalls dann, wenn keine konkret im Raum stehenden Erkenntnisse zum Beispiel wegen eines besonders schweren Krankheitsverlaufs mit Verdacht auf eine Virusmutation eingewendet werden, sollten sich die Gemeinschaftsinteressen gegen grundrechtlich geschützte Widersprüche Einzelner nicht mehr durchsetzen, da ohne ein akutes gesellschaftliches Anliegen die – von den Angehörigen geltend gemachte – Freiheit des Einzelnen im Umgang mit seinem früheren Körper überwiegt.

Ferner müssen zum Teil anwendbare weniger eingriffsintensive Maßnahmen von den zuständigen Behörden bedacht werden, die ähnliche Forschungsergebnisse erzielen könnten. In Betracht kommen etwa minimal-invasive Eingriffe in Form punktueller Biopsieentnahmen oder bildgebender Verfahren zur Diagnostik und Gewebesammlung[69]. Wirken die milderen Maßnahmen aber nur ergänzend zur Leichenöffnung[70], indem bspw. gefundene Ergebnisse mikrobiologisch/virologisch erhärtet werden, oder können die Alternativmaßnahmen aufgrund hoher, nicht an jedem Ort verfügbarer technischer Voraussetzungen nicht durchgeführt werden, dürfen sie der Anordnung der Amtsobduktion nicht im Wege stehen. Der insoweit nötige Vergleich zwischen den verschiedenen Erkenntnismitteln muss in Abhängigkeit von der konkreten Forschungshypothese erfolgen und den Beurteilungsspielraum der Ermittelnden achten.

## IV. Handlungsbedarf in insbesondere rechtlicher Hinsicht

Nach den bisherigen Darlegungen liegt in § 25 Abs. 4 S. 2 IfSG zwar durchaus eine Regelung vor, welche eine *pandemic preparedness* im Kontext der Obduktion nach Art des sog. Hamburger Weges gestattet. Dennoch lässt sich vor dem Hintergrund des praktischen Leerlaufs der Norm nicht festhalten, dass Deutschland auf die nächste Pandemie hinsichtlich der behördlichen Anordnung postmortaler Reihenuntersuchungen vorbereitet wäre. Es besteht ein offenbar beträchtlicher Handlungsbedarf.

Insoweit ist zunächst unverzichtbar, die tatsächliche Handlungsfähigkeit der oft überforderten Gesundheitsämter insbesondere personell und technisch zu steigern. Zudem müssen die Kenntnisse über das praktische Potential und die rechtliche Praktikabilität der Amtsobduktion verbessert werden, damit auch § 25 Abs. 4 S. 2 IfSG auf Basis seiner tatsächlich naheliegenden Auslegung ein „Arbeitsparagraph" werden kann.

Wenn das Ziel in einem gerade krisenfesten Umgang mit § 25 Abs. 4 S. 2 IfSG besteht, sollte jedoch auch das

69) *Kniep et al.*, Radiologe, 2020, 60, 927 ff.; s. auch das „postmortal imaging and biopsy program" des Instituts für Pathologie der Technischen Universität München unter federführender Leitung von PD Dr. med. habil. *Julia Slotta-Huspenina*, dazu *Boor et. al.*, Pathologe, 2021, 40, 197, 202 f. Zu den Vor- und Nachteilen postmortaler bildgebender Diagnostik bereits vor der Pandemie, *v. Stillfried et. al.*, Pathologe, 2017, 38, 412 ff.

70) Der lediglich ergänzende Einsatz wurde bspw. für die minimal-invasive, ultraschallgesteuerte Autopsie bei COVID-19- Obduktionen herausgestellt, *Boor et. al.*, Pathologe, 2021, 40, 197, 206.

Recht Fortschritte machen, damit in der nächsten gefährlichen Pandemie z. B. auch ein „Kölner Weg“ ernsthaft in Frage kommt, mit dem sich Erkenntnisse zugunsten der beiderseits akut bedrohten Güter Gesundheit und Freiheit mehren lassen. Hierzu sollte das IfSG klarer gefasst werden. Die erhobene Anwendungsunsicherheit sollte nicht länger den Rückgriff auf die gesetzlich legitime Amtsobduktion unterbinden. Überdies wird die eher stiefmütterliche und wenig klare bisherige Regelung auch ihrem Gegenstand, der eingriffsintensiven Obduktion, nicht gerecht.

### *1. Regelungskern: Klarstellung der Reichweite*

Zuallererst sollte sich die zu fordernde Zwecksetzung der Amtsobduktion nicht nur aus der Gesamtsystematik des IfSG oder aus Materialien ergeben. Der Umstand, dass die Norm über die Verbreitungseindämmung in unmittelbaren Einzelfällen hinaus anwendbar ist, muss angesichts der Bedeutung für besonders gefährliche Pandemien eine klarstellende Verankerung im Wortlaut erfahren, die durch Regelbeispiele für hinreichende Erkenntnisgewinne erzielt werden kann.

Ferner sollte das Gesetz die notwendige Koppelung des weiteren Anwendungsbereichs an den Pandemieverlauf und den Erkenntnisstand in einer geeigneten Weise verdeutlichen. Dies ist zur Begrenzung der bei einer zwangsweisen Anordnung erheblichen Grundrechtseingriffe geboten, damit die Trennlinie zu der befürchteten abstrakten Erkenntnissuche nicht überschritten wird. In diesem Sinne ist die Rückkopplung an eine gefährliche, akute Pandemielage erforderlich, welche zu ihrer Eindämmung dringend epidemiologische Erkenntnisse verlangt. Insoweit ist es vorzugswürdig, aber bisher nicht vollends möglich[71], den Umfang durchführbarer Obduktionen sowie die Bedeutung eines etwaigen Widerspruchs an die im IfSG vorfindlichen Qualifizierungen epidemischer Lagen anzulehnen, indem etwa die konkrete Gefahr eines sich dynamisch ausbreitenden neuartigen Erregers oder Mutationstypus aufgegriffen wird (vgl. § 28a Abs. 8 S. 2 IfSG in der bis 1. 10. 2022 geltenden Fassung[72]), welche kontaktunabhängiger Eingriffe bedarf, um den allgemeinen Gesundheitsschutz hinreichend sicherzustellen.

### *2. Weiterer Regelungsbedarf*

Zusätzlich sollten weitere Regelungen getroffen werden:

a) Reichweite und Verfahren

Die dargelegte Option, mittlerweile vorhandene mildere alternative Mittel zu ergreifen, sollte der nicht notwendig medizinisch versierten Anordnungsbehörde schon mit der Norm selbst verdeutlicht werden. Die milderen Alternativen sind, vergleichbar zu den Untersuchungsmöglichkeiten an Lebenden (vgl. § 25 Abs. 3 IfSG), beispielhaft auszudifferenzieren. Dabei sollten eingriffsintensive Alternativen wie bspw. die Biopsieentnahme aufgrund ihrer ebenfalls erhöhten Grundrechtssensibilität bei einer Durchführung gegen den Willen der Betroffenen ebenfalls an eine akute Pandemielage rückgekoppelt werden.

Ferner sollte den Rechten der Hinterbliebenen in insbesondere prozeduraler Hinsicht Ausdruck verliehen werden. Eine zwingende Benachrichtigung durch das Gesundheitsamt über die geplante Obduktion sowie, wenn dies den Erkenntnisgewinn nicht erheblich einschränkt, eine Anhörung der Angehörigen sollten vorgeschrieben werden, um nicht zuletzt eine lediglich einseitige Novellierung zu vermeiden[73]. Im Wege der Information und Anhörung der Angehörigen kann und sollte zumindest versucht werden, Bedenken auszuräumen und damit den Zwangscharakter zu mindern, indem die epidemiologische Notwendigkeit der Amtsobduktion hinreichend erläutert wird. Ähnlich wie bei einem dringlich erforderlichen ärztlichen Eingriff sind zeitliche Einschränkungen hierbei unvermeidbar, um den Erfolg der Untersuchung nicht zu gefährden.

Infolge der Rückkopplung der zwangsweisen Anordnung an eine vorliegende akute epidemische Lage besteht zudem Raum, in einem späteren Pandemiestadium, in dem ausreichendes Wissen gesammelt oder untersuchte Annahmen entkräftet werden konnten, Widersprüchen gegen die Durchführung zu entsprechen, wenn nicht im Einzelfall spezielle Erkenntnisgewinne die einzelne Sektion induzieren. Um die Bedenken bzgl. der Amtsobduktion zu zerstreuen, dürfte es überdies sinnvoll sein, die Selbstverständlichkeit ausdrücklich festzuhalten, dass ein pietätvoller Umgang mit dem Leichnam gewährleistet wird und der Obduktionsdurchführung immanent innewohnt. Die Mitteilung des Obduktionsergebnisses im Anschluss kann nach unserer praktischen Erfahrung überdies für die Trauerbewältigung der Angehörigen besonders bedeutsam sein. Sie sollte daher von vornherein vorgesehen werden.

b) Regelung der Organ- und Probenentnahme und die weitere Verwendung erhobener Daten und Proben

Die Amtsobduktion wird ihr Erkenntnispotential nur ausschöpfen können, wenn bei ihrer Durchführung Organe und spezifische Gewebeproben entnommen werden können. Dies ist zum einen notwendig, um mikroskopische und laborchemische Folgeuntersuchungen extrakorporal durchführen zu können. Zum anderen ist es für den Forschungsdiskurs unerlässlich, gewonnene Proben zu asservieren und auszutauschen, um Vergleichswerte zu bestimmen und Ergebnisse gemeinsam zu evaluieren[74]. Die Gewebeentnahme und Verwahrung ist damit integraler Bestandteil der Wissensmehrung durch die Amtsobduktion[75], und mithin nicht von einer gesonderten Zustimmung der Betroffenen abhängig zu machen. Dies sollte vergleichbar zu landesgesetzlichen Vorschriften (vgl. § 10 Abs. 1 S. 2 BestattG NRW[76] oder § 6 Abs. 2, § 11 Abs 2 oder § 14 Abs. 3 Hmb. SektionG[77]) und der gerichtlichen Sektion (§ 87 StPO i. V. mit mit Nr. 35 RiStBV[78]) im IfSG klargestellt werden. Eindeutiger Konsens unter Pathologen und Rechtsmedizinern

71) Der Ende 2021 zu beobachtende eher politisch-mutwillige Umgang mit dem Begriff der epidemischen Lage von nationaler Tragweite macht diesen Anschluss problematisch, s. zur Kritik *Schwarz*, COVuR 2021, 706 f.; *Kingreen*, in: *Huster/Kingreen*, Hdb. InfSR, 2. Aufl. 2022, Kap. 1, Rdnr. 123, weshalb zusätzlich eine eigenständige Umschreibung der gemeinten zugespitzten Situation erforderlich ist. § 28a Abs. 8 S. 2 IfSG in der bis 1. 10. 2022 geltenden Fassung bot insoweit alternative Anregungen.

72) Eine vollständige Übernahme des Rechtsbegriffs der „konkreten Gefahr einer sich dynamisch ausbreitenden Infektionslage“, ist aufgrund dessen Ausrichtung auf die SARS-CoV-2-Pandemie nicht zu verfolgen. Die Rückbindung an eine akute epidemische Lage muss entwicklungsoffen gestaltet sein, welches sich auch anhand der späteren Aufhebung der Begrifflichkeit durch die Anpassung des IfSG für die Herbst Schutzmaßnahmen 2022 (vgl. die in Fn. 66 genannte Gesetzesanpassung) einmal mehr bewahrheitet.

73) Ein Anhörungsrecht wird für die Hinterbliebenen regulär nicht schon aus § 28 (L-)VwVfG erwachsen, da diese als mittelbare Drittbeeinträchtigte keine Beteiligten des Verwaltungsverfahrens i. S. v. § 13 VwVfG sind, s. nur *Raumsauer*, in: *Kopp/Raumsauer*, VwVfG, 23. Aufl. 2022, § 28, Rdnr. 1. Darüber hinaus gehende Ansätze dürften eine Rechtsposition zumindest nicht hinreichend klar vermitteln.

74) Vgl. statt vieler *Bundesärztekammer* in Stellungnahme zur „Autopsie“ – Langfassung v. 26. 8. 2005, S. 38.

75) *Dettmeyer/Madea*, Rechtsmedizin 2004, 14, 85, 90; OLG München, NJW 1976, 1805; *Rixen*, ZRP 2001, 374, 375.

76) „Die Obduktion umfasst auch die Entnahme von Organen und Gewebeteilen sowie deren Aufbewahrung“.

77) Die genannten Paragraphen gestatten explizit im Rahmen der Durchführung der Sektion die Entnahme von Organ- und Gewebeteilen.

78) Nach der – nicht gesetzlichen – Vorschrift Nr. 35 RiStBV gestattet die StPO die Entnahme von Körperflüssigkeiten und Geweben sowie anderen Leichenteilen, wenn dies zur Feststellung von Todesursache und Todeszeitpunkt erforderlich ist, vgl. auch *Lippert*, MedR 2001, 406, 409.

hierbei ist, die Probenmenge jeweils auf das gerade notwendige kleinste Volumen zu begrenzen.

Nicht zuletzt vor dem Hintergrund der Diskussion zur erstmaligen oder veränderten postmortalen Verwendung rechtmäßig erhobener Körpersubstanzen zu Forschungszwecken[79] sollte die Regelung zudem die Verwendung der entnommenen Proben und der erhobenen Daten adressieren. Klarzustellen ist jedenfalls, dass das gewonnene Material unter der Kontrolle der für die rechtmäßige Erhebung zuständigen Stellen weiter für die Zwecke des Infektionsschutzes verwendet werden darf. In Anlehnung an den bereits zu nichtmenschlichen Erhebungen geltenden § 16 Abs. 3 IfSG sollte insbesondere die weitere anonymisierte Verarbeitung von Proben, die aus einer Obduktion gewonnen werden konnten, zulässig sein. Gerade die systematische Sammlung und Auswertung der Proben werden die Evaluation der bereits auf einer beträchtlichen Anordnungsschwelle beruhenden Erkenntnisse erst ermöglichen. Darüber hinaus wird auch nach dem Ende der akuten Infektionslage jedenfalls die weitere Nutzung zugunsten des Infektionsschutzes ohne weitere materielle Hürden zuzulassen sein. Dies gilt, weil die Zweckbindung nicht substanziell verändert wird und die Weiterverwendung einer bereits erhobenen Probe im Kontext des lediglich postmortalen Schutzes eine niedrigere Eingriffsschwelle gestattet. Im Ergebnis führt dies die für Untersuchungsmaterial von Lebenden in § 25 Abs. 3 S. 3 IfSG implementierte Regelung fort. Die Verwendung zu anderen Forschungszwecken würde den nun vermehrt umstrittenen allgemeinen Maßstäben folgen, die in dieser auf das IfSG bezogenen Arbeit nicht gleichsam nebenbei behandelt und ebenso wenig allein im IfSG zu regeln sind.

Bei alledem wird sich nicht sagen lassen, dass entsprechende Regelungen schon über den Verweis des § 25 Abs. 2 IfSG auf Teile des § 16 IfSG getroffen worden seien. Da erst § 25 Abs. 3 IfSG gesondert die Ermächtigung zur Entnahme von Untersuchungsmaterial von Menschen regelt, ist § 16 Abs. 2 S. 1 IfSG ebenso wie der akzessorische § 16 Abs. 3 IfSG nicht dahin zu interpretieren, dass er menschliche Proben betrifft[80]. Mindestens besteht der Bedarf, die Zulässigkeit der Probenentnahme nicht länger einem mehrdeutigen und mehrstufigen Binnenverweis zu überlassen. Auch hinsichtlich der Zweckbindung ist eine wie in § 25 Abs. 3 S. 3 IfSG vorgesehene konkretere Regelung einer Vorschrift vorzuziehen, die allein auf die zuständige Behörde bezogen ist (s. § 16 Abs. 1 S. 2 Halbs. 2 IfSG).

c) Weitere Durchführungsbestimmungen

Im Übrigen sollte das IfSG an der bisher über § 25 Abs. 2 IfSG vermittelten Anwendbarkeit der Durchführungsbestimmungen in § 16 Abs. 2, 5 und 8 IfSG festhalten. So vermag der Rechtsanwender vor allem bei außerklinischen Todesfällen zu erkennen, dass der Gewahrsamsinhaber des Leichnams verpflichtet ist, den Beauftragten der zuständigen Behörde und des Gesundheitsamtes den Zugang zum Verstorbenen zu ermöglichen (§ 16 Abs. 2 S. 2 IfSG)[81]. Weiter könnte § 16 Abs. 5 IfSG praktische Relevanz entfalten, wenn der Gewahrsamsinhaber geschäftsunfähig oder in der Geschäftsfähigkeit beschränkt sein sollte. Zudem sollte bei der Neuregelung sichergestellt werden, dass der Widerspruch oder die Anfechtungsklage gegen eine Amtsobduktion keine aufschiebende Wirkung entfalten kann (§ 16 Abs. 8 IfSG), da modernste ultrastrukturelle Untersuchungsmethoden frisches Gewebe der Verstorbenen für suffiziente Analysen voraussetzen. Innerhalb einer akuten Pandemielage kann nur damit ohne Verzögerung sichergestellt werden, dass die vom Gesetz legitimierte, aber nicht zeitlich flexible Erkenntnismehrung erreicht werden kann.

### *3. Regelungsort und konkreter Vorschlag*

Zuletzt ist der Regelungsort der Novellierung zu bestimmen. Insoweit erscheint die Einführung eines § 25a IfSG gegenüber einer deutlich ausgeweiteten Fassung des § 25 IfSG vorzugswürdig. Eine eigenständige Norm hebt zunächst die Bedeutung der Obduktion als besonderes Erkenntnisinstrument für den Rechtsanwender hervor. Nachdem die in § 25 IfSG vorgenommenen Änderungen, die das Anordnungsverhalten fördern sollten, in der Praxis nur sporadisch zur Kenntnis genommen worden sind, sollte die Obduktion prominenter herausgestellt werden. Überdies wird eine eigenständige Regelung vor allem der Grundrechtsdimension der zwangsweisen Anordnung einer Obduktion gerecht, die regelmäßig mit den Verstorbenen und ihren Angehörigen sogleich mehrere Personengruppen betrifft.

Auch systematisch ist es angezeigt, die Amtsobduktion von anderen Ermittlungsbefugnissen zu entkoppeln. Diese stehen weiterhin vor allem sinnvoll im Bezug zu einzelnen Infektionsketten und richten sich primär an lebende Personen. Die Amtsobduktion hingegen wird ihrem Gefahrenabwehr- und Freiheitsschutzpotential erst gerecht, wenn sie unter Umständen einen Erkenntnisgewinn allgemeinerer Natur über das Virus bzw. die Krankheit anstreben darf, wobei die vom Gesetzgeber etablierte Erstreckung auf die pandemieabschirmende Prophylaxe übernommen werden soll. Dieses erweiterte Anwendungsfeld kann in einer gesonderten Norm besser adressiert werden, zumal auch die Obduktionen bei vermeintlich impfstoff-induzierten Todesfällen nicht mehr systemfremd in den § 25 IfSG einzubinden wären. Ferner wäre die Ausrichtung am aktuellen Pandemieverlauf ein Novum in § 25 IfSG, das sich nicht ohne Weiteres auf andere Ermittlungsmaßnahmen übertragen ließe. Es erscheint vielmehr sinnvoll, alle postmortalen Untersuchungsmaßnahmen unter besonderer Hervorhebung der Obduktion in eine andere Norm zu überführen, um somit die Ermittlungsbefugnisse gegenüber Lebenden und Verstorbenen in Korrelation zu den unterschiedlichen berührten Grundrechten differenziert zu regeln[82].

Ein Gesetzestext, der insbesondere die dargestellten Ansätze aufgreift, könnte nach alledem wie folgt lauten:

§ 25a IfSG
Postmortale Untersuchungen

(1) [1] Um wichtige infektionsepidemiologische Erkenntnisse für Maßnahmen zur Bekämpfung übertragbarer Krankheiten zu gewinnen, kann das Gesundheitsamt erforderliche postmortale Untersuchungen Verstorbener anstellen. [2] Erforderlich sind Untersuchungen insbesondere über Art, Ursache und Ansteckungsquelle sowie Ausbreitung der Krankheit. [3] Gleiches gilt für die

79) S. m.w.N. für einen strikten Zustimmungsbedarf *Spranger/Böse/Jansen/Paeffgen/Verrel*, MedR 2022, 8 ff. in Auseinandersetzung mit der Stellungnahme der ZEKO v. 20. 2. 2003; vgl. auch *Dettmeyer/Madea*, Rechtsmedizin 2004, 14, 85, 92 f.; offener für sog. Altmaterial und bei einer Anonymisierung *Taupitz/Schreiber*, Bundesgesundheitsbl. 2016, 304, 306; *Lippert*, MedR 2001, 406, 410; bei deutlich überwiegenden Forschungszwecken *Nitz/Dierks*, MedR 2002, 400 ff. Ferner wird der Rückgriff auf eine mutmaßliche Einwilligung diskutiert, *Taupitz/Schreiber* ebd., 304, 306; *Freund/Weiss*, MedR 2004, 315, 317.

80) *Zwanziger*, in: *Eckart/Winkelmüller*, BeckOK IfSG, Stand 1. 7. 2022, § 16, Rdnr. 45. Die Vorschrift soll vielmehr der Untersuchung von verdächtigen Lebensmitteln dienen, BT-Dr. 8/2468, S. 19.

81) Da darin auch eine Einschränkung der Unverletzlichkeit der Wohnung nach Art. 13 Abs. 1 GG liegen kann, muss zugleich auf § 16 Abs. 4 IfSG verwiesen werden, um dem Zitiergebot nachzukommen.

82) So muss bspw. bei den betroffenen Grundrechten durch postmortale Untersuchungsmaßnahmen das Zitiergebot Art. 19 Abs. 1 S. 2 GG nicht mehr eingehalten werden. Nach ständiger Rspr. des BVerfG ist dieses nur bei solchen Grundrechten zu befolgen, die einem ausdrücklich formulierten Einschränkungsvorbehalt unterliegen; auch vorbehaltslos gewährleistete Grundrechte bedürfen danach keiner Zitation durch den Gesetzgeber, s. nur BVerfGE 83, 130, 154 m.w.N.; s. näher *Dreier*, in: *Dreier*, GG, 3. Aufl. 2013, Art. 19 Abs. 1, Rdnrn. 25 f. S. im Übrigen Fn. 81.

Aufklärung eines Todesfalles, der durch eine Schutzimpfung oder eine andere Maßnahme der spezifischen Prophylaxe eingetreten sein könnte.

(2) [1]Die Gewahrsamsinhaber des Leichnams können durch das Gesundheitsamt verpflichtet werden, erforderliche Untersuchungen oder geringfügige Entnahmen von Gewebematerial am Leichnam durch die Ärzte des Gesundheitsamtes und dessen ärztliche Beauftragte zu gestatten, insbesondere die äußere Leichenschau, Abstriche von Haut und Schleimhäuten sowie Verfahren der bildgebenden Diagnostik. [2]Unter den Voraussetzungen von Abs. 3 S. 4–7 können zudem Biopsieentnahmen oder andere vergleichbar eingriffsintensive Maßnahmen von den in S. 1 beschriebenen Personen vorgenommen werden.

(3) [1]Die zuständige Behörde soll gegenüber dem Gewahrsamsinhaber die innere Leichenschau anordnen, wenn dies vom Gesundheitsamt für erforderlich gehalten wird. [2]Mildere Untersuchungsmaßnahmen nach Abs. 2 sind vorrangig in Erwägung zu ziehen, soweit sie im Wesentlichen gleiche Erkenntnisse versprechen. [3]Die innere Leichenschau umfasst die erforderliche Entnahme von Organen und Gewebeteilen sowie deren Aufbewahrung. [4]Erforderlich ist die innere Leichenschau zur Bekämpfung übertragbarer Erkrankungen insbesondere, wenn

1. sich ergibt oder anzunehmen ist, dass der Verstorbene krank, krankheitsverdächtig, ansteckungsverdächtig oder Ausscheider war oder in Kontakt zu einer Person stand, die diese Eigenschaften erfüllt,
2. Erkenntnisgewinne bezüglich des Verbreitungsweges und der Ansteckungsgefahr des Krankheitserregers,
3. Erkenntnisgewinne hinsichtlich des Verlaufs der Infektionskrankheit,
4. Erkenntnisgewinne bezüglich möglicher Ursachen für Langzeitschäden,
5. Erkenntnisgewinne beim Verdacht auf Erregermutationen sowie hinsichtlich deren Beeinflussung des Krankheitsverlaufs,
6. sonstige Erkenntnisgewinne hinsichtlich eines bisher unbekannten Krankheitserregers, welche eine zu erwartende oder bereits erfolgte epidemische oder pandemische Ausbreitung verhindern oder eindämmen können, erzielt werden können oder
7. Erkenntnisgewinne erwartet werden, welche die Entwicklung oder Erforschung der Wirkungskraft einer Schutzimpfung oder anderer Maßnahmen der spezifischen Prophylaxe fördern.

[5]Untersuchungen, die wie zum Beispiel nach Abs. 3 S. 4 Nr. 2–7 über eine Verbreitungseindämmung im unmittelbaren Einzelfall hinausgehen, setzen die konkrete Gefahr einer sich dynamisch ausbreitenden Infektionslage voraus. [6]Dies gilt insbesondere, wenn großflächig Untersuchungen angeordnet werden oder Anordnungen gegen den Willen des Verstorbenen oder seiner Hinterbliebenen erfolgen. [7]Die konkrete Gefahr einer sich dynamisch ausbreitenden Infektionslage nach Satz 5 besteht, wenn der Deutsche Bundestag eine epidemische Notlage von nationaler Tragweite nach § 5 Abs. 1 S. 1 festgestellt hat oder eine Epidemie sonst zum Schutz der Gesundheit Eindämmungsmaßnahmen nahelegt, die über die Unterbrechung konkreter einzelner Infektionsketten hinausgehen und

Nr. 1 ein unzureichender Kenntnisstand über einen neuartigen Erreger in Verbindung mit einer zu erwartenden niedrigen gesellschaftlichen Immunität besteht, oder

Nr. 2 sich eine bisher unbekannte Erregervariante ausbreitet, die eine signifikant höhere Pathogenität vermuten lässt.

(4) [1]Die Angehörigen des Verstorbenen sind in den Fällen des Abs. 3 und Abs. 2 S. 2 zu benachrichtigen und, soweit keine entgegenstehenden dringenden Gründe vorliegen, anzuhören.

(5) [1]Bei Vornahme postmortaler Untersuchungen sind die Anforderungen eines pietätvollen Umgangs mit dem Leichnam stets zu berücksichtigen. [2]Den Angehörigen des Verstorbenen ist das Ergebnis der Untersuchungen mitzuteilen.

(6) [1]Das von der zuständigen Behörde oder deren ärztlichen Beauftragten erhobene Untersuchungsmaterial sowie anderweitige personenbezogene Daten dürfen nur von der zuständigen Behörde sowie dem Gesundheitsamt oder deren ärztlichen Beauftragten für Zwecke dieses Gesetzes verarbeitet werden. [2]Soweit es eine epidemische Lage erfordert, kann das Gesundheitsamt Anordnungen über die Übergabe des Untersuchungsmaterials zum Zwecke der Untersuchung und Verwahrung an Institute des öffentlichen Gesundheitsdienstes oder andere vom Bund oder Land zu bestimmende Einrichtungen oder Forschungsprojekte treffen.

(7) [1]Für die Durchführung der Untersuchungen gilt § 16 Abs. 2, 4, 5 und 8 entsprechend.

Im Zuge der Novellierung wäre § 25 Abs. 4 IfSG zu streichen. Der neue Absatz 4 wäre der frühere Absatz 5 der Norm.

## V. Fazit

Das Infektionsschutzrecht weist mit § 25 Abs. 4 S. 2 IfSG durchaus eine Regelung auf, die Amtsobduktionen in verhältnismäßiger Form als Mittel der Bekämpfung ausgeprägter Pandemien gestattet. Soll die tatsächliche Nutzung dieses Mittels aber nicht weiterhin auf der zufälligen Initiative Einzelner beruhen, sind tatsächliche und insbesondere rechtliche Reformen anzuregen. Die grundrechtssensible Amtsobduktion sollte im IfSG z. B. in einem § 25a IfSG eine nähere Regelung erfahren, um diese Option differenziert zu bekräftigen und zugleich sinnvoll verfahrensmäßig abzustützen. Auf diesem Wege kann Deutschland die nun schmerzlich als notwendig erfahrene *pandemic preparedness* nennenswert und überzeugend fördern und Handlungssicherheit schaffen. Es kann damit die Hoffnung nähren, dass wissenschaftliche Erkenntnisse abermals den Zielen des Gesundheits- und Freiheitsschutzes werden dienen können.

**Open Access.** Dieser Artikel wird unter der Creative Commons Namensnennung 4.0 International Lizenz veröffentlicht, welche die Nutzung, Vervielfältigung, Bearbeitung, Verbreitung und Wiedergabe in jeglichem Medium und Format erlaubt, sofern Sie den/die ursprünglichen Autor(en) und die Quelle ordnungsgemäß nennen, einen Link zur Creative Commons Lizenz beifügen und angeben, ob Änderungen vorgenommen wurden.
Die in diesem Artikel enthaltenen Bilder und sonstiges Drittmaterial unterliegen ebenfalls der genannten Creative Commons Lizenz, sofern sich aus der Abbildungslegende nichts anderes ergibt. Sofern das betreffende Material nicht unter der genannten Creative Commons Lizenz steht und die betreffende Handlung nicht nach gesetzlichen Vorschriften erlaubt ist, ist für die oben aufgeführten Weiterverwendungen des Materials die Einwilligung des jeweiligen Rechteinhabers einzuholen.
Weitere Details zur Lizenz entnehmen Sie bitte der Lizenzinformation auf http://creativecommons.org/licenses/by/4.0/deed.de.

Funding

Open access funding enabled and organized by Projekt DEAL.

